# Association between the Use of Quantitative Sensory Testing and Conditioned Pain Modulation and the Prescription of Medication and Interventional Procedures in Children with Chronic Pain Conditions

**DOI:** 10.3390/children9081157

**Published:** 2022-08-02

**Authors:** Alice Bruneau, Catherine E. Ferland, Rafael Pérez-Medina-Carballo, Marta Somaini, Nada Mohamed, Michele Curatolo, Jean A. Ouellet, Pablo Ingelmo

**Affiliations:** 1 Division of Experimental Medicine, McGill University, Montreal, QC H3A 0G4, Canada; alice.bruneau@mail.mcgill.ca; 2 Department of orthopedic surgery, Shriners Hospitals for Children-Canada, Montreal, QC H4A 0A9, Canada; catherine.ferland@mcgill.ca (C.E.F.); jaouellet@hotmail.com (J.A.O.); 3 Edwards Family Interdisciplinary Center for Complex Pain, Montreal Children’s Hospital, Montreal, QC H4A 3J1, Canada; ma.somaini@gmail.com (M.S.); nada.mohamed@muhc.mcgill.ca (N.M.); 4 Department of Anesthesia, McGill University, Montreal, QC H3A 3J1, Canada; 5 Alan Edward Centre for Research on Pain, Montreal, QC H3A 0G1, Canada; 6 Integrated Program in Neurosciences, McGill University, Montreal, QC H3A 0G4, Canada; rafael.perez@douglas.mcgill.ca; 7 Department of Anesthesia, Niguarda Ca’Granda Hospital, 20162 Milan, Italy; 8 Department of Anesthesiology and Pain Medicine, University of Washington, Seattle, WA 98195, USA; curatolo@uw.edu; 9 Research Institute, McGill University Health Center, Montreal, QC H3G 3J1, Canada

**Keywords:** quantitative sensory testing, conditioned pain modulation, chronic pain, pharmacotherapy

## Abstract

The evidence supporting the use of pharmacological treatments in pediatric chronic pain is limited. Quantitative sensory testing (QST) and conditioned pain modulation evaluation (CPM) provide information on pain phenotype, which may help clinicians to tailor the treatment. This retrospective study aimed to evaluate the association between the use of QST/CPM phenotyping on the selection of the treatment for children with chronic pain conditions. We retrospectively analyzed the medical records of 208 female patients (mean age 15 ± 2 years) enrolled in an outpatient interdisciplinary pediatric complex pain center. Pain phenotype information (QST/CPM) of 106 patients was available to the prescribing physician. The records of 102 age- and sex-matched patients without QST/CPM were used as controls. The primary endpoint was the proportion of medications and interventions prescribed. The secondary endpoint was the duration of treatment. The QST/CPM group received less opioids (7% vs. 28%, respectively, *p* < 0.001), less anticonvulsants (6% vs. 25%, *p* < 0.001), and less interventional treatments (29% vs. 44%, *p* = 0.03) than controls. Patients with an optimal CPM result tended to be prescribed fewer antidepressants (2% vs. 18%, *p* = 0.01), and patients with signs of allodynia and/or temporal summation tended to be prescribed fewer NSAIDs (57% vs. 78%, *p* = 0.04). There was no difference in the duration of the treatments between the groups. QST/CPM testing appears to provide more targeted therapeutic options resulting in the overall drop in polypharmacy and reduced use of interventional treatments while remaining at least as effective as the standard of care.

## 1. Introduction

Chronic pain in children and adolescents has a prevalence of 10–25% and often persists into adulthood [1,2,3,4]. There is currently no evidence supporting the use of pharmacological treatments such as antidepressants, anticonvulsants, non-steroidal anti-inflammatory drugs (NSAIDs), or opioids for the treatment of chronic non-cancer pain in children or adolescents [5,6]. There is also a lack of evidence for the safety profiles of these medications, and the incidence of adverse effects remains largely unknown [6]. Nevertheless, poly-pharmacological treatments and interventions are regularly prescribed to pediatric patients for chronic pain [6].

Chronic pain can be dichotomized into chronic primary pain and chronic secondary pain. Chronic primary pain is defined as “pain in one or more anatomical regions that persists or recurs for longer than 3 months and is associated with significant emotional distress or functional disability and that cannot be better accounted for by another chronic pain condition”. Examples of chronic primary pain include widespread chronic pain, complex regional pain syndrome, chronic primary visceral pain, chronic primary musculoskeletal pain, chronic primary headache, and orofacial pain [7]. Chronic secondary pain syndromes are linked to other diseases as the underlying cause, for which pain can initially be regarded as a symptom. Examples of chronic secondary pain include chronic secondary musculoskeletal pain, chronic neuropathic pain, chronic postsurgical and post-traumatic pain, chronic secondary headache, and orofacial pain [7]. Improving the drug-selection process by providing predictive values of treatment efficacy and safety could help clinicians deliver improved individualized care to patients [8,9].

Quantitative sensory testing (QST) and the conditioned pain modulation (CPM) evaluation are tools that can be used in the research setting to predict treatment responses in various pain conditions [9,10]. QST/CPM evaluations identify alterations in sensory processing of mechanical, thermal, and noxious stimuli [11,12,13] and may suggest an underlying pain mechanism in children and adolescents [14,15,16,17]. Previous studies suggested that QST/CPM phenotype information could help clinicians select the appropriate pharmacological treatment for chronic pain [18,19].

We hypothesized that patients with a QST/CPM assessment could be prescribed medical treatments (pharmacological or interventional) in different proportions compared to those patients that have not been assessed using QST/CPM. This hypothesis was built on the premise that a treatment plan based on the sensory phenotype allows physicians to tailor the treatment without deterioration of patient outcomes while reducing the number of medications prescribed. Therefore, we aimed to describe and compare the prescription patterns of patients with and without the QST/CPM assessment. The main objective of this retrospective study was to evaluate if the use of QST/CPM assessments in a pediatric chronic pain interdisciplinary program has an impact on the selection of pharmacological or interventional treatment. The secondary objective of the study was to evaluate and compare the duration of the treatment.

## 2. Materials and Methods

### 2.1. Patients

Patients aged 9 to 17 years referred to the ambulatory program of the Edwards Family Interdisciplinary Center for Complex Pain were included in the study. We used the Center for Complex Pain database to identify potential candidates for the analysis. The initial selection included patients between 9 and 17 years old, reporting persistent or recurrent non-cancer pain for at least three months from September 2015 to October 2018. The second screening included patients with complete psychosocial and physical function evaluation and complete information regarding the treatment and cause of the end of treatment. As female individuals represented over 83% of the sample, we excluded male subjects to avoid gender variations during the analysis. In addition, we excluded patients who would not be candidates for QST/CPM testing (i.e., younger than seven years old, developmental delay, etc.), patients who did not understand English or French, and patients with a cancer diagnosis. Finally, patients who had a QST/CPM assessment before their first evaluation were matched according to age and diagnosis using a computerized randomization method to patients who did not have a QST/CPM assessment (“standard of care control group”) in a 1:1 ratio. The Center for Complex Pain has used a comprehensive QST protocol to assess mechanisms of pain as well as a CPM protocol to evaluate the endogenous descending pain inhibitory control of patients before the initiation of a treatment since early 2016. The evaluation was systematically offered to patients starting in September 2017.

Chronic pain conditions were categorized retrospectively for the purpose of this study using the ICD-11 classification of chronic pain using the categories of (1) chronic primary pain and (2) chronic secondary pain [7].

### 2.2. Interdisciplinary Evaluation (Standard of Care)

The interdisciplinary outpatient program of the Center for Complex Pain focuses on optimizing physical and psychological function, normalizing sleep and social function, and increasing levels of activity while assisting with the management of the pain. The core team at each evaluation includes a nurse clinician, psychologist, social worker, physiotherapist, clinical fellow, and a pain physician. The clinical team of the Center for Complex Pain remained constant during the study period, excluding the change of one social worker, one nurse clinician, and the rotation of clinical fellows. At baseline, the team evaluates sleep quality, physical function, and psychological function using the Pittsburgh Sleep Quality Index [20] (PSQI), the Functional Disability Inventory [21,22] (FDI), and the Revised Child Anxiety and Depression Scale [23] (RCADS), respectively. After an interdisciplinary simultaneous interview with the team and their parent(s), the patient undergoes a physical examination (clinical fellow, physician, and physiotherapist) that includes a detailed neurological exam with particular attention to changes in sensations. Pain is assessed consistently using the Numerical Rating Scale (where 0 represents no pain, 10 represents the worst pain imaginable) during patient evaluations with the interdisciplinary team. The pain was assessed at rest and with activity. The clinicians report the presence and distribution of hyperalgesia, allodynia, dysesthesia, loss of sensation, and any other specific changes on the standard neurological exam.

### 2.3. QST/CPM Evaluation Protocol

The full evaluation is termed QST/CPM evaluation but may be abbreviated to “QST” only for short in the tables/figures. The QST protocol was based on previous comprehensive studies and includes assessments of mechanical detection threshold, vibration detection threshold, dynamic mechanical allodynia, pain pressure threshold, heat pain threshold, and mechanical pain summation. QST/CPM evaluations were offered as a clinical service at the first appointment at the clinic, and parents were not present in any of the evaluations. The procedures were standardized and performed in triplicates by a single QST/CPM technician. The results were evaluated and compared to reference values from the literature, when available, with respect to protocol and test sites [15,24]. Tests were performed on both control sites and painful locations when possible. The endogenous descending pain inhibitory pathway was evaluated using a CPM paradigm of tonic thermal stimulations [11,25,26].

The pain pressure threshold (PPT) was measured using a hand-held digital pressure algometer (Jtech) with a display in Newtons. If the PPT was significantly below the lower bound of the 95% CI of the reference values at a control site (masseter, thenar muscle, or ball of the foot, as described by Blankenburg and colleagues in a group of healthy children and adolescents [15]) or significantly lower (difference of at least 30%) compared to a same-subject’s contralateral site in the instance of unilateral pain, deep-tissue pressure pain sensitivity was reported. A greater pressure pain sensitivity indicates enhanced mechanical sensitivity [15].

The presence of dynamic mechanical allodynia was reported using a standardized brush (Somedic SENSELab—Brush-05). Allodynia is defined by the IASP as “pain due to a stimulus that does not normally provoke pain” [27]. On a mechanistic level, dynamic allodynia is proposed as a lack of inhibition of excitatory crosstalk between sensory modalities (touch and pain) by interneurons in the spinal dorsal horn [28]. In other words, there is a failure to separate the input from Aβ-fibers (touch) and nociceptive-specific neurons [28,29].

The presence of temporal summation was evaluated both mechanically and thermally. Mechanical temporal summation was evaluated using the difference in the self-reported pain between one and ten stimulations with a Neuropen (Owen Mumford) with a standardized 40 g Neurotip at a rate of 1 per second. Mechanical temporal summation was evaluated at a control site (masseter, thenar muscle, or ball of foot) as well as the painful site within the individual, but not to external reference values due to the difference in the tool used. Thermal temporal summation was measured on the medial forearm during a constant heat stimulus over a period of 2 min at a pre-determined temperature self-reported to cause ≥ 5/10 pain and interpreted as the difference in pain intensity between the numerical rating score at 60 s and at 120 s of the test [25]. If a patient was not able to tolerate the 2 min test, the temperature was decreased by 0.5 °C and repeated until they were able to tolerate it for 2 min. For both temporal summation tests, a significant increase of >2/10 in pain rating was considered a positive result based on Initiative on Methods, Measurement, and Pain and Assessment in Clinical Trials (IMMPACT) recommendations for clinically important differences in pain intensity [30]. All thermal testing was performed using the Medoc QSense apparatus and a computerized visual analog scale (CoVAS). Mechanistically, temporal summation, also called wind-up, may reflect an increase in the excitatory postsynaptic potentials in response to repeated C-fiber stimulation [28,29]. Studies suggest that temporal summation is stronger in individuals with primary chronic pain (including fibromyalgia) compared to normal controls [31,32].

Mechanical detection threshold and vibration detection thresholds were investigated using Von Frey Filaments and a 64 Hz tuning fork (Rydel-Seiffer), respectively, and compared to reference values for the zygomatic bone, processus styloideus ulnae, or malleolus internus [15]. These measures were used as additional information regarding the integrity of the Aβ-fibers as complementary information to suggest the possibility of deafferentation pain.

The endogenous descending pain inhibitory pathway control was quantitatively evaluated using the CPM paradigm developed by Marchand and colleagues [25] and simplified to be used in younger patients [33]. The paradigm consists of the difference in continuous pain rating during two tonic thermal heat pain stimulations on the right medial forearm separated by a cold-water conditioning stimulus consisting of a left forearm immersion of 2 min at 12 °C. The thermal heat component was performed using the Medoc QSense and a computerized visual analog scale (CoVAS). The two-minute painful thermal stimulation temperature was pre-determined as the temperature at which the patient experienced a self-reported pain of ≥5/10, as described in previous studies [11,26]. The efficacy of the CPM test was categorized as efficient, suboptimal, or inefficient. An efficient CPM score corresponded to a pain reduction of 30% or more, whereas an inefficient CPM score corresponded to a pain reduction of less than 10% or pain facilitation [11,26]. The suboptimal CPM category was included as a conservative buffer to allow for a margin of error (pain reduction between 11% and 29%) [11,25,26]. The numerical value of the CPM was also reported for each patient. An inefficient CPM result is suggested to reflect an incapacity to trigger a proper endogenous pain inhibition [11,26,34,35,36,37].

### 2.4. Proposed Treatments

After the physical examination, the team proposed an individualized treatment program for the patient and their family, including non-pharmacological as well as pharmacological and interventional treatments. The cost of the evaluations, treatment, and follow-up provided by the Center for Complex Pain is entirely covered by the Quebec public health system and by unrestricted grants from the Montreal Children’s Hospital Foundation, the Louise and Alan Edwards Foundation, and the Pathy Family Foundation.

The multidisciplinary program included cognitive behavioral therapy (individual and groups) and outpatient physiotherapy once a week or twice a month, depending on the patient’s needs. The medical treatment varied based on the information available during the clinical evaluations. When the QST/CPM evaluation results were not available, the clinicians chose the medical treatment using treatment guidelines for specific conditions (i.e., headache, widespread chronic pain, functional abdominal pain, complex regional pain syndrome, etc.). In addition, they selected the treatment based on the findings of the physical examination (i.e., allodynia, hyperalgesia, loss of sensation, etc.) or on the type of pain (neuropathic pain, inflammatory pain, sensitization, etc.).

When clinicians had the results of the QST/CPM evaluation, they used a simplified protocol in addition to the findings of the physical examination (Table 1). The treatment protocol was based on mechanistic considerations and adapted from previous studies on adults and children with chronic pain conditions [19,35,38,39,40,41,42,43,44,45,46,47,48,49,50]. In cases where all three QST/CPM phenotypes were present (phenotypes A, B, and C), the most common order of proposal of pharmacological agents was: (1) pharmacological treatment for phenotype B, (2) for phenotype C, (3) for phenotype A. Interventional procedures were indicated in case of localized pain even if the source was expected to be on deep nociceptors such as muscles. Interventional procedures such as central blocks were only proposed after other avenues were explored because patients would need to be admitted to the ward. When cases were more complex, for example, when patients presented with multiple diagnoses or mixed physical findings, the clinicians used their clinical experience to determine the best treatment course possible. All the data gathered from the auto-evaluation and the initial evaluation and follow-ups were prospectively documented in the Center for Complex Pain database and transferred to the patient’s electronic chart.

### 2.5. Primary Endpoint

The primary outcome measure was the number and type of medical treatments prescribed by the team to treat pain. Medical treatments were grouped into six categories: antidepressants; opioids; non-steroidal anti-inflammatories (NSAIDs), including diclofenac topical cream; anticonvulsants; other drugs (including mainly magnesium supplements, melatonin supplements, oral clonidine and topical non-NSAID creams); and interventional treatments. Most interventional treatments consisted of ultrasound-guided peripheral nerve blocks with very few ablative procedures (mainly radiofrequency), as previously described by Vega and colleagues [51].

### 2.6. Secondary Outcome Measures

The initial treatments prescribed to patients with primary and secondary pain conditions were independently analyzed. We also analyzed the duration of treatment as an independent variable. We defined the duration of treatment as the time between the first ambulatory evaluation and the completion of the treatment. Patients were considered to have completed the treatment when they reported minimal (NRS < 3/10) or no pain, were not taking pain medication, and were presenting normal physical and role functioning (school attendance). Other reasons for early termination of the treatment included transfer to another specialty, transfer to adult care on their 18th birthday, or loss to follow-up.

### 2.7. Statistical Analysis

Descriptive statistics were computed to describe the patient sample included in the study. An independent samples *t*-test for the difference of means of continuous variables or Mann–Whitney’s U test for non-parametric variables was performed to compare baseline characteristics between groups in terms of mood, function, pain intensity, and sleep. In order to address the main hypothesis, binary logistic regression analysis was performed to compare the number of patients prescribed different drug treatments (dependent variable) between the QST/CPM evaluation group (“QST group”) and standard treatment group (independent variable). The results were reported for pooled data, by diagnosis category, and by QST/CPM results. Finally, plots of Kaplan–Meier estimates with log-rank tests were produced to compare the duration of treatment of patients (time between first ambulatory visit and completion of treatment). Reasons for missing data included transfer to another specialty, transfer to adult care on their 18th birthday, or loss to follow-up. In those cases, the data were entered as “censored” in the Kaplan–Meier model and log-rank test. The significance level was established at 0.05. Bonferroni corrections for multiple comparisons were applied where appropriate. Statistics were performed using IBM SPSS Statistics for Windows, Version 24.0. Armonk, NY, USA: IBM Corp.

## 3. Results

### 3.1. Clinical Characteristics

A total of 208 female patients between the ages of 9 and 17 years were included in the analysis. One hundred and eight patients had a chronic primary pain diagnosis (50% in the standard control group and 51% in QST/CPM group), and 100 had a chronic secondary pain diagnosis. There were no differences between the QST/CPM group (*n* = 106) and the standard treatment group (*n* = 102) with regard to age, diagnosis, pain intensity, sleep quality, function, and mood at baseline (Table 2). The patients had a wide variety of diagnoses, including inflammatory diseases such as Elher Danlos syndrome and juvenile arthritis, chronic migraines, post-traumatic and postsurgical pain, complex regional pain syndrome, widespread chronic pain, and amplified musculoskeletal pain syndrome. The most common diagnosis was chronic back pain. At baseline, 77% of the patients experienced moderate to severe physical disability measured with FDI, and 89% experienced poor sleep quality evaluated with the PSQI questionnaire. The great majority of patients (98%) received physiotherapy and cognitive behavioral therapy (85%) with no difference between groups.

**Figure 1:**
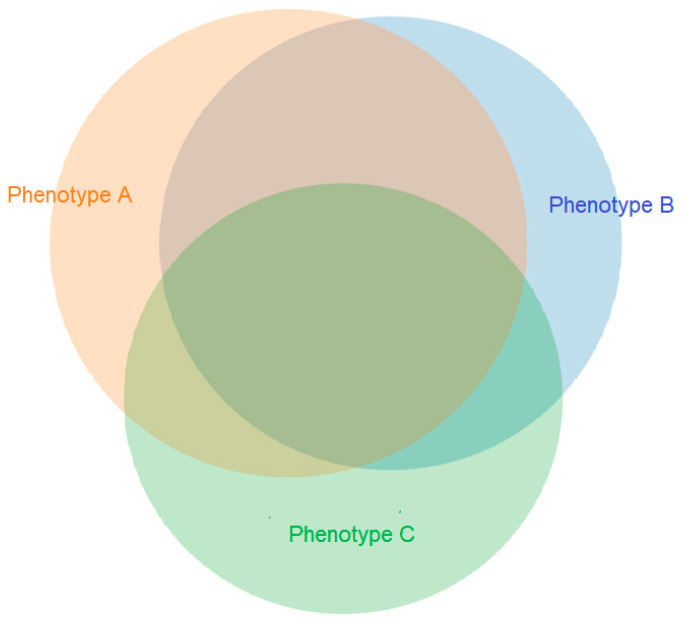
Distribution of QST and CPM phenotypes (*n* = 106). Phenotype A, Presence of allodynia and/or temporal summation; Phenotype B, presence of pressure pain sensitivity; Phenotype C, CPM inefficient or suboptimal endogenous inhibitory pain response. Only 5 patients had none of the pain phenotypes present (results within normal limits).

The availability of the QST/CPM testing results was based on the availability of the testing room and technician at the time of the appointment, which can be considered overall as a random factor. All patients who completed QST/CPM evaluation had complete QST/CPM results available, with no patient dropping out because of intolerance to pain. Among patients in the QST/CPM group, 65% presented with dynamic mechanical allodynia and/or temporal summation (phenotype A), 61% presented with sub-threshold pressure pain sensitivity (phenotype B), and 55% had a suboptimal or inefficient endogenous pain modulation (phenotype C). A total of 26% presented with all three abnormal sensory processing phenotypes (Figure 1).

### 3.2. Primary Outcome Measures

**Figure 2:**
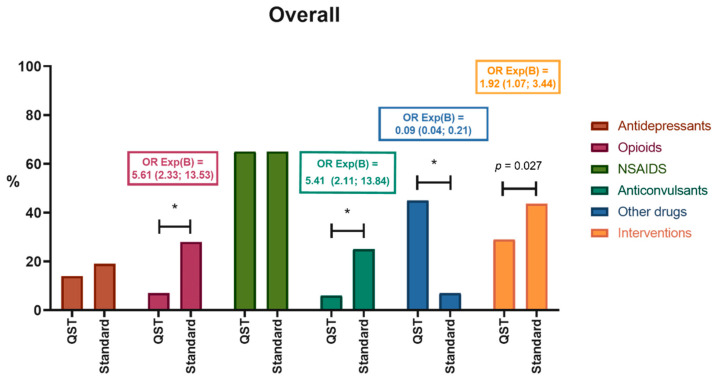
Medical treatment prescribed at the initial visit for the overall sample, comparing patients with standard assessment and QST/CPM assessment. * *p* < 0.008, using Bonferroni correction. Odds ratios (OR) are described.

There were significant differences in the number and type of medications prescribed when patients were tested with the QST/CPM compared with patients without testing. Patients in the QST/CPM group received fewer opioids, fewer anticonvulsants, and more frequently other drugs (including mainly magnesium supplements 25%, melatonin supplements 25%, oral clonidine 13%, and topical non-NSAID creams 19%). Patients in the QST/CPM group also tended to receive less interventional treatments (Figure 2).

**Figure 3:**
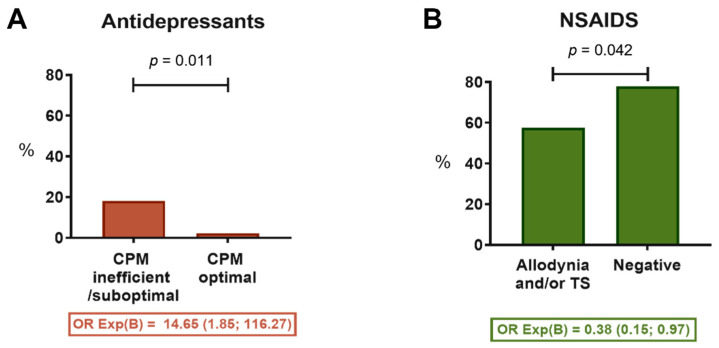
Comparison of medical treatment prescribed at the initial visit specific to QST/CPM recommendations. (**A**) The proportion of patients with a prescription for antidepressants was greater among those with an inefficient or suboptimal endogenous inhibitory pain response; (**B**) The proportion of patients with a prescription of NSAIDs was smaller among those who showed positive signs of allodynia and/or temporal summation (TS). Odds ratios (OR) are described.

Patients in the QST/CPM group were prescribed a smaller number of medications. Patients from the standard group were prescribed on average 1.4 medications, whereas QST/CPM patients were prescribed on average 0.9 medications (*p* < 0.001). Patients with an efficient endogenous pain inhibitory response were prescribed fewer antidepressants (2% vs. 18%; *p* = 0.011); however, after Bonferroni correction for multiple comparisons (significance level of 0.008), this result was not significant. Patients with a positive sign of allodynia and/or temporal summation were prescribed fewer NSAIDs (57% vs. 78%; *p* = 0.042); however, after Bonferroni correction (significance level of 0.008), this result was not significant (Figure 3).

### 3.3. Secondary Outcome Measures

**Figure 4:**
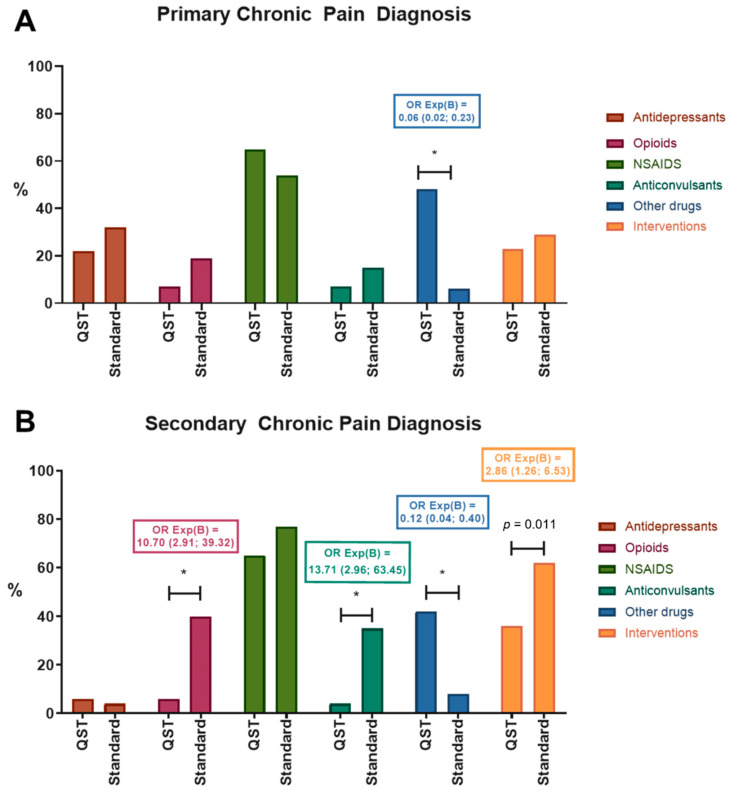
Medical treatment prescribed at the initial visit by chronic pain category and QST group. (**A**) Chronic primary pain diagnosis (chronic primary widespread pain, chronic regional pain syndrome, chronic primary visceral pain, chronic primary musculoskeletal pain, chronic primary headache, and orofacial pain); (**B**) Chronic secondary pain diagnosis (chronic secondary musculoskeletal pain, chronic neuropathic pain, chronic postsurgical and post-traumatic pain, chronic secondary headache, and orofacial pain). * *p* < 0.008, using Bonferroni correction. Odds ratios (OR) are described.

Among patients with chronic primary pain, there was no significant difference between the two groups in the prescription of antidepressants, NSAIDs, opioids, anticonvulsants, and interventional treatment (Figure 4A). However, the clinical team prescribed more other drugs when QST/CPM results were available. Patients with chronic secondary pain were prescribed fewer opioids and anticonvulsants and tended to receive fewer interventional procedures when the QST/CPM results were available. Like primary pain patients, they received more other drugs. There were no significant differences in the prescription of antidepressants and NSAIDs in patients with chronic secondary pain (Figure 4B).

**Figure 5:**
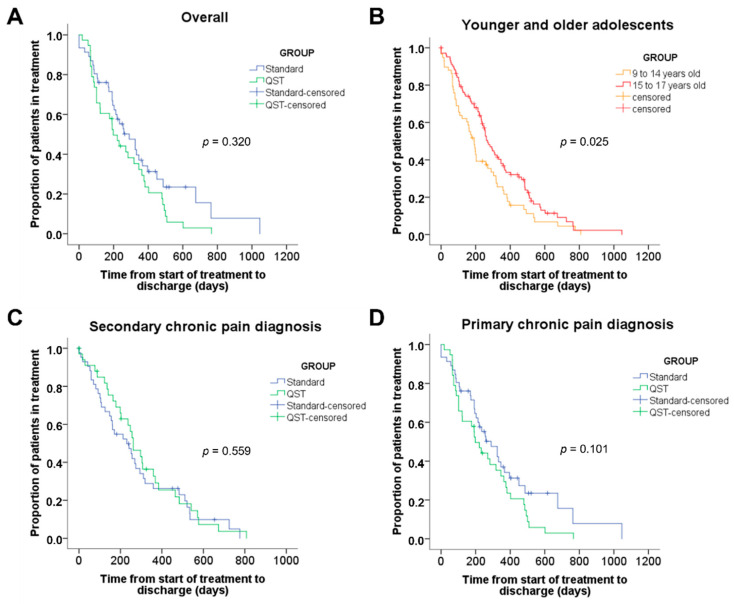
Kaplan–Meier plots of the duration of treatment of patients at the clinic expressed in days. Patients who left the clinic due to other reasons than discharge were censored. Such reasons may include transfer to another specialty (e.g., surgery, psychiatry), loss of follow-up, or transfer to adult treatment when patients reached 18 years old. (**A**) Overall comparison of patients with standard assessment and QST/CPM assessment (n.s.); (**B**) Comparison of younger patients (9–14 years old) and older patients (15–17 years old), (*p* = 0.025); (**C**) Comparison of patients with standard assessment and QST/CPM among patients with primary pain diagnosis (n.s.); (**D**) Secondary chronic pain diagnosis (n.s.). Using a Bonferroni correction for multiple comparisons (significance level of *p* = 0.125), none of the results were significant. However, in panel B, there appears to be a trend for younger patients to complete treatment more rapidly than older patients.

There was no significant difference in duration of treatment between the QST/CPM and standard assessment control group, regardless of diagnosis (Figure 5). Overall, 76% of patients completed their treatment, reporting NRS < 3/10, without taking pain medication and having normal physical functioning and normal school attendance. More than 60% of patients spent less than one year in the program (mean treatment duration = 257 days, 95% CI [223 to 291], median = 213 days). Patients entering the clinic between 9 and 14 years old appeared to have completed treatment faster than older patients (15–17 years old) on the Kaplan–Meier plot; however, by using a Bonferroni correction for multiple comparisons on the log-rank test, this result was found to be not significant (*p* = 0.025). Twenty-five individuals were censored (i.e., considered “missing data”) from the treatment duration model because they had left the clinic due to a transfer to adult care on their 18th birthday (*n* = 15), transferred to another specialty (*n* = 3), or loss to follow-up (*n* = 7).

The efficacy and side effects of each treatment were not assessed in this retrospective chart review. If at follow-up, a patient reported no improvement in pain or significant side effects, the clinical team offered an alternative treatment and continued offering individualized care to each patient, including psychotherapy and physiotherapy.

## 4. Discussion

In this study, we examined the effect of using an exhaustive pain phenotyping protocol based on mechanical and thermal QST and a CPM evaluation in an interdisciplinary center for complex pain. A single physician was in charge of the medical treatment of the patients included in this study, along with four rotating fellows under his supervision, thus limiting differences in physician preferences of treatment modalities. When QST/CPM evaluations were available, the physicians prescribed less opioids and other medications and tended to perform fewer interventional procedures, especially among patients with chronic secondary pain. Overall, the use of the QST/CPM evaluations did not change the length of the treatment.

Patients who demonstrated an efficient endogenous pain inhibitory control tended to be prescribed fewer antidepressants. Moreover, patients with dynamic mechanical allodynia and/or temporal summation tended to be prescribed fewer NSAIDs. The clinicians in this study followed treatment protocols based on previous studies and recommendations for the treatment of adults and children [19,34,35,38,39,40,41,42,43,44,45,46,47,48,49,50]. Longitudinal studies are needed to evaluate the efficacy and safety of the pharmacological treatments used in adolescents and children with chronic pain conditions.

Eccleston and colleagues stated that “given the widespread use of off-license prescribing in pediatric pain medicine and the absence of data concerning pain and its management, we need to focus on benefits of drugs as well as harms and harm reduction” [6]. Our mechanistic treatment protocol based on the QST/CPM analysis was associated with a meaningful reduction in the number of prescribed medications (0.5 medications per patient) and the use of interventional treatments. However, the proportion of patients with minimal or no pain, not taking pain medication, and reporting normal physical functioning leaving the clinic and the duration of treatment for the patients were similar between groups. Future randomized and longitudinal studies should evaluate the potential influence of QST/CPM evaluations on harm reduction.

A trend was observed where children between 7 and 14 years old seemed to recover faster compared with those between 15 and 18 years. This might be explained by the temporality of the progression of chronic pain conditions. Individuals who suffer from pain for a prolonged period without treatment may experience long-term neuroadaptations that further promote pain and disability [2,29,52].

Female individuals represented over 83% of the patients in the clinic, which is reflective of the higher prevalence of chronic pain conditions in females in the population [1]. Due to sex differences in pain experiences [53] and the few male patients with QST/CPM evaluation in the database for sex-specific analysis, the current analysis included female patients only.

The present study presented limitations. The study consisted of a retrospective analysis of female patients at a single institution, and it is uncertain how generalizable the current results are. Future multicenter randomized studies should evaluate the weight of QST/CPM evaluations in the efficacy of treatments and in the prevention of the disability associated with complex pain conditions. The pharmacological and interventional treatments are only a part of the different therapeutic options offered within an interdisciplinary program. As part of a comprehensive multidimensional pain treatment program, it is not easy to interpret the weight of potential benefits of individual therapies. However, a meaningful reduction in the number of medications prescribed was observed after the implementation of the QST/CPM evaluations, which is considered a positive effect due to the reduction in polypharmacy. Side effects were not analyzed specifically in this study. The QST/CPM protocol was performed on appointment, without regard to pain episodes. This means patients may have received the QST/CPM evaluation during a period when their pain was less intense or more intense.

Although we found some encouraging results in our study, the use of a comprehensive QST/CPM protocol remains complex and increases the costs of the consultations. Moreover, pain phenotyping protocols need to be simplified to be used in daily clinical practice. The QST/CPM protocol used in this study required between 75 and 90 min to complete and document. This research group is working on the creation of a simplified version for future studies.

## 5. Conclusions

The sensory assessment may guide clinicians in selecting different treatment options for their patients. A mechanistic approach may reduce the use of potentially unnecessary treatments without affecting the clinical outcomes of children with complex pain conditions.

## Figures and Tables

**Table 1:** Treatment recommendations according to QST/CPM phenotype.

QST/CPM Phenotype	Treatment
**Phenotype A: Allodynia and/or Temporal Summation**	
First line	Gabapentinoids
Second line	Ketamine (IV or oral), opioids
**Phenotype B: Pain Pressure Sensitivity**	
First line	NSAIDs, topical treatments, interventional treatments for well-localized pain
Second line	Lidocaine IV infusions
**Phenotype C: Inefficient or Suboptimal Conditioned Pain Modulation**	
First line	Tricyclic antidepressants, Oral Clonidine
Second line	Selective Serotonin Reuptake Inhibitors

**Table 2:** Patients’ characteristics at baseline (*n* = 208).

	Standard *n* = 102		QST/CPM *n* = 106		*p*-Value
	Mean	SD	Mean	SD	
Age	14.7	2.2	14.5	2.2	0.48
FDI	20.4	10.9	22.7	11.6	0.15
PSQI	9.1	3.8	9.3	4.4	0.80
RCADS General anxiety	43.8	9.6	45.3	11.4	0.31
RCADS Depression	55.0	13.6	53.0	16.0	0.36
Pain (0–10 VAS)	5.2	2.1	5.5	1.9	0.28

## Data Availability

The data presented in this study are available on request from the corresponding author.
